# Cheek—Does It Pay?

**Published:** 1881-10

**Authors:** Ben. H. Pratt


### THE BISTOURY.

Vol. XVIII. ELMIRA, N. Y., OCTOBER, 1881. No. 3.

### Contributions.

### CHEEK--DOES IT PAY?

BEN. H. PRATT.

We sometimes hastily covet that which we do not really desire to possess. This is true of character traits rather than of material things.—Among traits of character there is, possibly, nothing that seems so admirable as cheek. This is more universally coveted than any other single characteristic among men. And yet I question whether he who covets cheek would accept the alleged boon were he assured that it must be his for the asking. One admires the man of infinite cheek, but is the admiration so aroused of a kind that we would like to inspire within the bosoms of our associates generally? I have pondered this matter of human cheek, animate brass, a good deal. There have been times when I would have given that which I esteem the most valuable of my alleged attainments in exchange for a modicum of cheek. It has seemed to me, many is the time, the most valuable of all earthly possessions; the talisman of success; the open sesame to all the portals that lead to honor, fame, wealth, distinction, preeminence in any field that the possessor may select. It has often seemed to be the sine qua non of on-getting in life, and without which nothing worth attaining is attainable. It apparently places the ignorant not only upon the plane of the cultured, but above it. No wonder that men have coveted cheek when it brings to the possessor so much that is seemingly desirable, and at such small cost of labor, thought, intellect.

But there is that which is more to be desired than honor, fame, wealth and distinction, especially when these are purchased with cheek. Having carefully weighed the question "Does Cheek Pay?" I am firmly convinced that it does not. I do not mean that it may not accumulate for the possessor much that we are wont to call the good things of life, but I do claim that the methods which cheek must employ in the getting of these things are such as to depreciate the getter in a degree for which the gain is not commensurate. It is possible that the man of cheek may never know that he is an object of a kind of admiration which is less desirable than pity. Undoubtedly it is a characteristic of the brassy man never to feel that he is different from other men save in what he terms smartness. I know that men of cheek are simply men of vast conceit, self assurance, confidence in themselves and with an utter disregard for the feelings and the sentiments of their fellows. Cheek is not only an index of selfishness but it is supreme selfishness itself. He that possesses cheek has no opinion of another's merit and will not admit that merit, outside of the smartness he calls cheek, has any existence. He mocks at what we call modesty, a retiring disposition, a tendency to be withdrawn from prominence. He scouts the notion that wisdom can be possessed without being paraded. He recks not that one can have something which he values too much to use upon all occasions. In fact, the man of cheek is essentially a boor. He does not know that there is any other use for that which he possesses save thereby to possess more, be that of which he is in pursuit what it may. He has no sense of the impression he is making upon others, and hence no notion of the price, in the estimate of his fellows, he is paying for what he believes he gains. He ignores everything that does not show. Like the vulgar, he arrays himself in his best upon all occasions, unconscious that he is false and tawdry to the beholder.

When one soberly compares cheek and wisdom, or contrasts them rather, there can be but one conclusion; and in the light of the fascinations which group themselves about the cheeky man, it behoves the wiser to warn youth against the arts and wiles of the brazen, lest cheek be cultivated for what it too often begets. I know that it is difficult to reason with any young man or young woman to the effect that there is special merit in modesty. There are too many apparent instances of the untruthfulness of the claim. I am aware that the allurements which the man of cheek can and does gather about his career are of a kind that destroy all our reasoning upon this subject. It is only by living a goodly number of years, and carefully noting the careers of the brazen, that one can surely satisfy himself that cheek does not pay. I do not know, therefore, that argument could avail one whit towards convincing any one that he will not be a gainer, all things considered, by encouraging and cultivating his assurance to its extreme limit. I do not know, either, that any number of examples given in illustration of the final outcome of the life of the cheeky man would prove of any service. We find men the same in every generation. Few are willing to take it for granted that the words of the older are always the words of wisdom. Almost every young man and woman prefers to test all the old saws of their elders, and are convinced only after they have made personal demonstrations of their truth. But there is some satisfaction in the thonght that there is a time coming in the life of nearly every young person when he will admit that he might have saved much time and anguish had he accepted, rather than sought to prove, the assertions of his elders. In no distinction is argument so weak, and in no direction has it such an array of truth upon its side as in this.

It matters little, however, whether one have or have not at hand argument to prove that cheek is not a desirable possession. Cheek is born in some people, and to whom it is not an inheritance it never comes. I do not believe that one can acquire cheek. I may possibly be modified some by cultivation, but it cannot be inculcated nor can it be obliterated. Cheek will develop in the child, if possessed. If not possessed, no teaching and no study can beget it. It may be clumsily counterfeited, but the sham is readily detected. Nor can it be concealed, if possessed, no matter how thoroughly the possessor may have learned to despise it, or how much he may be ashamed to have it recognized. Cheek will assert itself despite all effort at suppression, and oftener to the possessor's harm than to his advantage.

Cheek never passes current for wisdom among the really wise, and it does not, in the long run, gain for him who has it more than modesty does. It may appear to, but it does not. Cheek is not the most enviable thing in the world. I have thought it to be such, but the illusion is gone. "He who blows not his own horn, for him his horn shall not be blowed" is a taking motto. But when I see how little the world is inclined to give attention to such blowing, and when I note how commiseratingly if not contemptuously people of sense regard the exhibition of assurance, and how seldom mankind generally is deceived by it, and how truly those whose opinions are worth having value merit modestly kept in the background until needed, then I have no hesitation as to where I want to see those whom I respect. Cheek does not deceive any but him who possesses it. Observe the cheeky man, and give ear to the opinions that are expressed on all sides concerning him. You may be dazzled, nay even dazed by the brilliancy with which he shines for the hour. You may envy him his prominence for the nonce, and wish you might be such as he, but go your way—ponder upon what you hear of him, and then choose whether you would take his place and endure his reputation for all that his cheek has gained, whether in material things or in his position among men.

The world reviles the man of cheek when his back is turned. He may have high place and yet not have high position among men. Men may fawn before him and flatter him, and yet most cordially despise him. Society measures him for all he is, not for anything he seems to be. It tires of him, recognizing the difference between the brazen assumption and the golden merit with a nicety that is scarcely credible. The success which attends the cheeky man is not a real success. We may admire it for a space, but it is not anything to be dwelt upon with any satisfaction. We may yield to the importunity of the man of cheek, and we may get out of his way and allow him to press toward that for which he aims, but it is rather to avoid the bore and to escape from contact with him than because we recognize any right he has to occupy the place he seeks. The world is said to give way before push and thrust, but it is no special credit to the pusher that the world does so give way. We avoid such men as we would avoid any other disagreeable thing, lest it may defile us. It is no credit to a mad bull that a street full of people scatters into places of safety when he appears. It is no credit to the pole-cat that no one cares to dispute its pathway. We respect our persons, oftentimes, more than we do our dignity; and we have the same feeling toward the man of infinite cheek—do not balk him because it is too disagreeable a duty to meddle with him. We would rather see him achieve what he undertakes than bother with preventing him, or demeaning ourselves by accepting him as our companion in the race. No, there's nothing really enviable in cheek, and the more one has of it the more he is to be avoided and pitied.

For the young, however, there is a glamour around the man of cheek that arouses admiration, then envy, then imitation. Is it not natural for men to desire to appear better than they are—to veneer their lives in the hope of deceiving the world into a belief that they are, all through, just what their outside appears to be? Therefore cheek, chicanery, deception of every sort, must need abound. The modest man often repines because he, with tenfold the ability that another possesses, is ever out of sight, while the brazen ignoramus is continually parading before the eyes of men and receiving their feigned adulation. Nor is there any sure hope that modest merit will ever achieve its deserts; and the only satisfaction the latter often has is in the mere consciousness of its possession. There is no comparison between contentment and the grandest achievement secured by imposition; and cheek is an index of the boldest imposition that man can practice. It is worth something to be able to look every man in the face and feel that you've nothing to be especially ashamed of—to feel that you'd rather meet the man you've had business and social relations with than avoid him—to feel that whatever he knows about you, he isn't taking you either for much more than you're worth or for a knave. The great distinguishing characteristic among men seems to be this: Those of one class are ever striving to make the world believe that they know more than they do know; those of the other are afraid the world will over-estimate and expect too much of them. The former are the cheeky, the latter the modest men. There are grades of each, and the individuals of each class vary widely in the possession of their characteristics, but it does not require any of us a great while to classify our acquaintance and place each one pretty surely in the niche he should occupy. The world is not easily fooled. There are fewer fools than men are apt to think exist. Cheek may deceive a few for a brief time, but it cannot long deceive any one save the poor creature that possesses it.

